# Effect of adding TiO_2_ nanoparticles on the SEM morphology and mechanical properties of conventional heat-cured acrylic resin

**DOI:** 10.15171/joddd.2019.036

**Published:** 2019-10-07

**Authors:** Elnaz Moslehifard, Mahmood Robati Anaraki, Saeed Shirkavand

**Affiliations:** ^1^Department of Prosthodontics, Dental and Periodontal Research Center, Tabriz University of Medical Sciences, Tabriz, Iran; ^2^Department of Prosthodontics, Faculty of Dentistry, Tabriz University of Medical Sciences, Tabriz, Iran; ^3^Department of Prosthodontics, Faculty of Dentistry, Urmia University of Medical Sciences, Urmia, Iran

**Keywords:** Polymethyl methacrylate, compressive strength, metal nanoparticles, flexural strength, nanocomposite, dental materials

## Abstract

***Background.*** The current study evaluated the compressive, flexural and impact strengths of heat-cured acrylic resins reinforced by TiO_2_ nanoparticles (NPs).

***Methods.*** TiO_2_ NPs were provided and characterized using scanning electron microscopy (SEM) to determine their morphology and crystalline structure. For three mechanical tests, 12 acrylic resin groups (n=9), totaling 108 specimens, were prepared using a special mold for each test, with TiO_2_ nanoparticle contents of 0, 0.5, 1 or 2 wt% in different groups. After curing, the compressive, flexural and impact strengths of the specimens were examined according to ISO 1567.

***Results.*** In the SEM and XRD study of TiO2 NPs, anatase was identified as the major crystalline phase followed by rutile (average particle size: 20.4 nm). SEM images showed that the nanocomposite with 1 wt% NPs had a more homogenized blend. 1 wt% TiO_2_ nanocomposite exhibited a higher, but non-significant, impact strength compared to the controls. ANOVA showed significant differences in the impact and flexural strengths between nanocomposites with various contents of TiO2 NPs.

***Conclusion.*** The nanocomposite with 1 wt% TiO2 NPs exhibited fewer micro-pores and micro-cracks in the SEM crosssections. A non-significant increase was also observed in the impact strength with TiO_2_ NPs at 1 wt%. Further increase in TiO_2_ NPs decreased both the impact and flexural strengths. The compressive strength of the heat-cured acrylic resin was not affected by the incorporation of NPs.

## Introduction

Polymethyl methacrylate (PMMA) is the material most commonly used for fabricating removable dentures, orthodontic appliances and some types of removable or fixed implant prostheses. The advantages of this material are esthetic, biocompatibility, availability and easy manipulation. However, the mechanical strength of PMMA is not adequate, and attempts have been made to improve its mechanical strength. For example, its flexural strength is relatively low,^1,2^ and in the clinical situation, several factors can cause PMMA failure, including occlusal disharmonies, overload, fatigue, mishandling and accidental impacts.

Various methods have been employed for improving the mechanical properties of PMMA, including the chemical correction of its polymeric structure with additives, such as polyethylene glycol dimethacrylate.^3^ Another useful method to improve the mechanical properties of acrylic resins include the addition of different fillers.^4-8^ Nanoparticles (NPs) are one type of such fillers. NPs, with at least in one dimension measure of <100 nm, have opened up new horizons to overcome the limitations of their traditional counterparts.^9^ Titanium dioxide (TiO_2_) NPs have excellent mechanical properties and are inexpensive, with titanium being the most abundant metal on earth, following aluminum, iron and magnesium. The superior mechanical properties make them one of the ideal additives to enhance the performance of polymeric materials.^10^ TiO_2_ NPs have been demonstrated to be a useful multifunctional material and are used in a wide variety of environmental applications, including water treatment and air purification.^11,12^ In addition, their high chemical stability, low cost and non-toxicity make them ideal as an alternative material for improving the antimicrobial properties.^13^ Metal oxide NPs, including TiO_2_ and Fe_2_O_3_, have been shown to be suitable additives for improving PMMA.^14^ Human cell growth on titanium used for dental implants has also been shown to improve through the formation of a nano-network surface oxide layer.^15^

The ultimate flexural strength of a material reflects its potential to resist catastrophic failure under a flexural load. Flexural strength of denture base resin is considered the primary mode of clinical failure.^16^ Impact strength (IS) is also a favorable property because it is a measure of the energy required to initiate and propagate a crack through the material. Thus, it can reflect the contact force needed to cause a fracture in a denture under situations such as accidental dropping. The compressive strength is another feature of PMMA that shows the resistance of the material against vertical static loads and is critical in the denture base that is compressed under heavy occlusal forces. The occurrence of fracture, observed in maxillary and mandibular removable prostheses, results in additional costs, as well as discomfort to patients, as they must be without dentures during the laboratory procedures required to repair or replace the broken denture.^17^ Previous studies have not clearly shown reinforcement of essential mechanical features of the acrylic resin with TiO_2_ NPs.^14,16,18^ This study investigated the effect of incorporation of TiO_2_ NPs on the acrylic resin properties, including its compressive, flexural and impact strengths.

## Methods

### Preparation of the Specimens

The PMMA used in this study was heat-cured acrylic resin (Ivoclar Vivadent, Schaan, Liechtenstein). TiO_2_ NPs were purchased from the Anataz TiO_2_, Nanosav, Tehran, Iran, with an average diameter of <25 nm. The characterization test was validated by x-ray diffraction (XRD) test (X’pert MPD, Philips, Eindhoven, the Netherlands). The grain size of the TiO_2_ NPs may be obtained from X-ray diffractogram by the Scherrer formula:^23^

D= 0.9 λ/βcosθ

where D is the grain size, λ is the wavelength, β is the peak width, and θ is the angle. This equation shows the reciprocal relation of the grain size (D) and the broadening of peaks (β) in the full width in the middle height.

Specimens with three concentrations of TiO_2_, including 0.5, 1, and 2 wt% in acrylic resin, were compared with specimens of 0 wt% TiO_2_. The form and dimensions of the specimens were selected according to ISO 1567 standard for comparing the compressive strength, flexural strength and impact strength with those of the control group.^19^ Nine specimens for each of the four concentrations and 36 specimens for each mechanical test were required, and a total of 108 specimens in the twelve study groups were prepared. The sample size (n=9) was determined by a pilot study. An adequate power (large effect size according to Cohen’s effect size statistics) at α=0.05 and a power of 0.878 was obtained for detecting statistically significant differences.

Preparation of TiO_2_-PMMA composite with TiO_2_ NPs was carried out as follows: TiO_2_ NPs were mixed separately with the powder of acrylic resin polymer in an amalgamator for 20 minutes to obtain three different composites with 0.5, 1 and 2 wt% TiO_2_ contents. Then, the mixed solid powder was manually blended with the resin monomer to obtain a homogenized mixture. To form the paste, it was packed into different steel molds, resulting in different specimen forms as the proposed mechanical test, using a vibrator to remove any air bubbles. Cylindrical specimens, measuring 25 mm in diameter and 38 mm length, were used for the compressive strength test. Rectangular bar specimens, measuring 2×2×20 mm, were used for the flexure strength test. Rectangular bars, measuring 0.364×0.364×2.962 inch with an 0.05-inch-depth crossing notch on 1/2 of its length were used for the impact strength test. After processing according to the manufacturer’s instructions, the specimens were removed from the molds, finished and polished, using silicon carbide papers (mesh numbers of 500–2000). The TiO_2_ NPs were characterized using SEM (SEM, VEGA/TESCAN Czech Republic) to study the morphology of the specimens.

### Mechanical Experiments

The compressive strength test was carried out by a testing machine (Dartech; Alfre J. Amsler&Co, Germany), where the fracture of specimens was shown on the machine monitor.^20^

In the flexural strength measurement, the specimens were mounted in an Instron universal testing machine (three-point loading and testing equipment). The load was applied at the center of the specimen with a cross-head speed of 1.50 mm/min and a span length of 40.00 mm. The maximum load before the fracture was calculated. The results were recorded through the three-point bending test. The flexural strength was calculated as follows:^21^

$$\sigma =\frac{3FL}{2b{\text{d}}^{2}}$$

where σ is stress, F is load/break at heat (N), L is the span of the specimen, b is the width, and d is thickness.

In impact strength (IS) test, before testing, the specimens were notched with a notching cutter (Notchvis; Ceast, Pianezza, Italy). The V-notches were cut at half the length of the bar-shaped specimens, across the width of the specimens with 0.8-mm depth, leaving an effective depth.^22^ The IS was evaluated by the Charpy impact tester (Resil 25R; Ceast), with the un-notched surface of the specimens facing the pendulum. The test was performed with 0.5 J pendulum and a 150º lifting angle. IS was expressed in kJ/m^2^, calculated as IS = EC/(h/bA), where EC is the corrected energy absorbed by breaking the test specimen, bA is the remaining thickness at the notch tip, and h is the specimen width.

### Statistical Analysis

After testing, the data were analyzed with statistical methods. The mean, average and mode in each group were calculated, and normal distribution curve was evaluated. Kolmogorov-Smirnov test was used to evaluate the normal distribution. Statistical analysis of the results for each test group was conducted using one-way ANOVA, followed by multiple comparison test (Scheffe’s test). Statistical significance was set at P<0.05.

## Results

**Figure 1:**
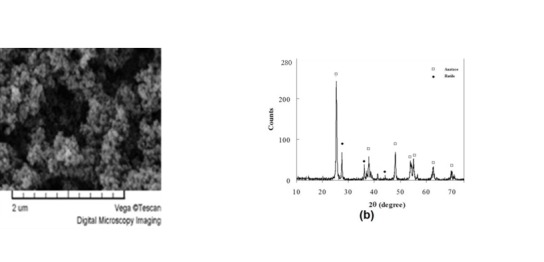
(a) SEM image; (b) XRD diagram of TiO_2_ nanoparticles.

Figure 1 shows the SEM images and XRD spectrum obtained from the titanium oxide NPs. Two crystalline polymorphs of titanium oxide with tetragonal crystal structures were present: (i) anatase and (ii) rutile.

**Figure 2:**
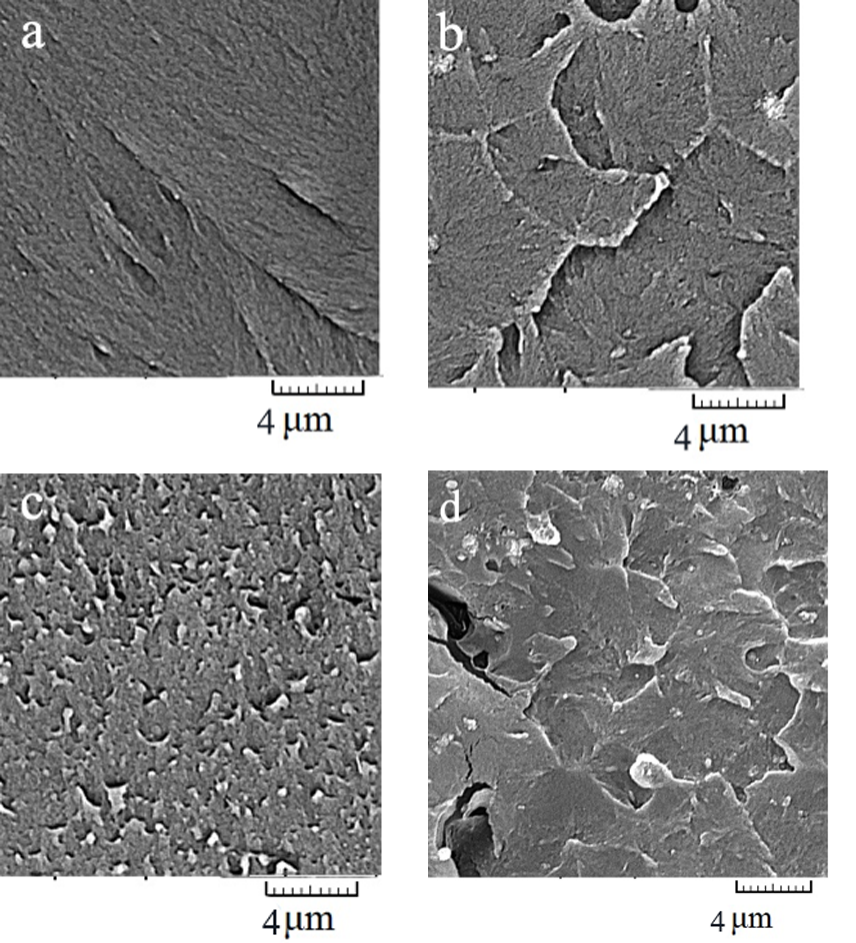
SEM images taken from the cross-section of nanocomposite containing TiO_2_ nanoparticles with (a) control sample (0 wt%), (b) 0.5 wt%, (c) 1 wt%, and (d) 2 wt%.

The microscopic morphology of nanocomposites in the cross-section is shown in Figure 2. TiO_2_ at 1 wt% had a more homogenized mixture compared to the other two nanocomposite groups. A comparison between Figures 2(c) and 2(d) revealed tiny cracks and micro-pores within the acrylic matrix with an increase in TiO_2_ NPs content from 1 wt% to 2 wt%.

**Figure 3:**
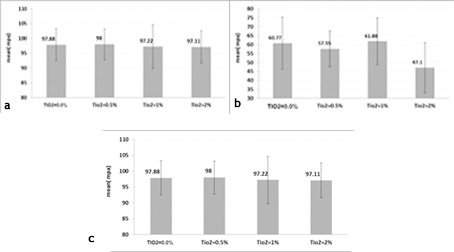
Means and standard deviations of (a) compressive, (b) flexural and (c) impact strengths calculated for all the specimens.

Figure 3(a) presents the mean ± SD of the compressive strength measured in all the groups. According to the results of ANOVA, there was no significant difference in the compressive strength between the three reinforced acrylic resin groups with different amounts of TiO_2_ and conventional acrylic resin.

Figure 3(b) presents the mean ± SD of flexural strengths. ANOVA showed a significant difference in the flexural strength between the nanocomposite group with 2 wt% NPs and the two other groups. An increase in the NPs content up to 1 wt% did not affect the flexural strength, whereas an increase to 2 wt% compromised the nanocomposite.

Figure 3(c) presents the mean ± SD of the impact strengths in the studied groups. ANOVA revealed significant differences in the mean impact strength between the groups (P<0.05). The results showed that incorporation of NPs at 0.5 and 2 wt% had negative effects on the impact strength of acrylic resin, while 1 wt% resulted in a non-significant increase in the impact strength.

## Discussion

As a common clinical problem, fractures in the acrylic denture base are considered a challenge in the current dental materials science. Therefore, several attempts have been made to improve the mechanical properties of PMMA, all of which can be summarized in three lines: replacing PMMA with an alternative material; chemically modifying it; reinforcing the PMMA with other materials like fibers or metals as fillers.^4,24-26^ In addition to improving the strength and preventing fracture in the denture base, another principal aim is to improve the stiffness in order to prevent resorption of the residual ridge, and also to overload the supporting implants, remaining teeth and surrounding structures.

This study aimed to investigate the possible effects of reinforcing compressive, flexural and impact strengths of the acrylic resin by adding TiO_2_ NPs as fillers at three concentrations of 0.5, 1 and 2 wt%.

The flexural strength is a principal factor in the resistance of the denture base to deformation and fracture. The results of the current study showed that the flexural strength decreased when the concentration of NPs was >1 wt%, possibly because NPs act as impurities in the nanocomposite structure. This is consistent with the results of a study by Ahmed et al.^10^ The results of the current study showed that while the flexural strength of the nanocomposite with 1 wt% NPs was higher than that with 2 wt% NPs, 1 wt% did not show a significant increase compared to 0 wt% NPs, a finding which was not consistent with previous studies showing a significant increase in the flexural strength with this percentage of NPs content.^27,28^ A higher flexural strength with 1 wt% NPs could be due to more homogenized blending of NPs in the nanocomposite as observed in the SEM study. The SEM images of 1 wt% also showed fewer micro-pores and micro-cracks in the cross-sections compared to other nanocomposites, particularly compared to the nanocomposite with 2 wt% NPs. Formation of cracks could originate from higher internal stress levels in the acrylic resin induced by more TiO_2_ particles added in the bulk polymer due to the substantial property of TiO_2_/polymer interface energy.^24^

The impact strength is another basic property of the resin base that prevents the fracture of dentures during accidental dropping. The impact strength results showed a significant increase for conventional acrylic resin modified by 1 wt% TiO_2_ NPs in comparison with the two other nanocomposites and no significant increase in comparison with the control of acrylic resin specimens. This result is different from previous studies reporting an increase in the impact strength by incorporating 1 wt% TiO_2_ NPs,^10,26^ possibly due to different techniques of experiments. Similar to what we observed in the flexural test, nanocomposite with 2 wt% NPs exhibited significantly decreased impact strength that can be related to excessive NPs and passing beyond the saturation capacity of acrylic resin, as more NPs cannot blend without agglomeration. Incorporation of excessive filler leads to an interruption in the resin matrix continuity and decreases the strength of the reinforced specimens. These findings are consistent with previous reports in the literature.^28-30^ As Karci et al^28^ reported, there was a decrease in the flexural strength values of PMMA with the incorporation of nanoparticles at 3‒5 wt%.

The compressive strength of the nanocomposite was not affected by the incorporation of TiO_2_ NPs up to 2 wt%, tested in this study, as there were no significant differences between the study groups.

Further studies are needed to investigate the effect of other nanomaterials on the mechanical and physical properties of PMMA.

## Conclusions

1. The nanocomposite with 1 wt% TiO_2_ NPs exhibited fewer micro-pores and micro-cracks at SEM cross-sections.
2. Incorporation of 1 wt% TiO_2_ resulted in a non-significant increase in the impact strength of the conventional heat-polymerized acrylic resin.
3. Incorporation of TiO_2_ filler did not enhance the compressive and flexural strengths of the conventional heat-polymerized acrylic resin.
4. Incorporation of TiO_2_ beyond 1 wt% can decrease the flexural and impact strengths of the conventional heat-polymerized acrylic resin.

## Competing Interests

The authors declare no competing interests with regards to the authorship and/or publication of this article.

## Acknowledgments

This article was written based on a dataset from an MSc thesis entitled “Comparison of the compressive strength, tensile strength, flexural strength and impact strength of acrylic resin reinforced with TiO_2_ nanoparticles and conventional acrylic resin” registered at Tabriz University of Medical Sciences, Faculty of Dentistry (reference number 160/T). The thesis was supported by the Vice Chancellor for Research at Tabriz University of Medical Sciences.

## Authors’ Contributions

SSH contributed to the definition of the intellectual content, literature search, experimental studies, experimental studies and data acquisition. EM contributed to the concept and design of the study, literature search, experimental studies, data acquisition, data analysis, statistical analysis, manuscript editing. MRA contributed to the definition of the intellectual content, literature search, data analysis, statistical analysis, manuscript preparation, as well as editing. All the authors have read and approved the final manuscript.

## Funding

Not applicable.

## Ethics Approval

Not applicable.
